# Bone Morphogenic Protein-2 (rhBMP2)-Loaded Silk Fibroin Scaffolds to Enhance the Osteoinductivity in Bone Tissue Engineering

**DOI:** 10.1186/s11671-017-2316-1

**Published:** 2017-10-25

**Authors:** Guang-Yu Du, Sheng-Wei He, Chuan-Xiu Sun, Li-Dong Mi

**Affiliations:** Department of Bone surgery, The Second Affiliated Hospital of Dalian Medical University, No.216, Shanzhong Street, Ganjing District, Dalian, 116031 Liaoning People’s Republic of China

**Keywords:** Tissue engineering, Silk fibroin scaffold, Recombinant human bone morphogenic protein-2 (rhBMP2), Osteoinductivity

## Abstract

There is an increasing demand for formulations of silk fibroin (SF) scaffolds in biomedical applications. SF was crosslinked via glutaraldehyde with osteoinductive recombinant human bone morphogenic protein-2 (rhBMP2) of different ratios viz. (i) 3% SF with no rhBMP2 (SF), (ii) 3% SF with equal amount of rhBMP2 (SF+BMP2), and (iii) 12% SF with 3% of rhBMP2 (4SF+BMP2), and these solutions were used in electrospinning-based fabrication of nanoscaffolds for evaluating increased osteoinductive potential of SF scaffolds with rhBMP2. Stress–strain relationship suggested there is no loss in mechanical strength of fibers with addition of rhBMP2, and mechanical strength of scaffold was improved with increase in concentration of SF. rhBMP2 association increased the water retention capacity of scaffold as evident from swelling studies. Viability of hMSCs was found to be higher in conjugated scaffolds, and scaffolds do not exhibit any cytotoxicity towards guest cells. Cells were found to have higher alkaline phosphatase activity in conjugated scaffolds under in vitro and in vivo conditions which establishes the increased osteoinductivity of the novel construct. The scaffolds were found to be effective for in vivo bone formation as well.

## Background

Regenerative capacity of bone allows repair of small bone fractures by itself. Bone is formed, followed by union, and finally, reconstruction of original shape and form of bone. However, this capacity is limited, and this creates a requirement of autografts or allografts for the treatment [1]. Allografting involves obtaining the bone from a separate donor that may cause immunological reaction. Autografting, where the bone is obtained from patient’s own body does not create immunological problems but is limited by the sufficient quantity of available bones [2–4].

Tissue engineering is being perceived as a potential technology to overcome the immunological limitation of allografting and autografting. With tissue engineering, specialized cells like human mesenchymal stem cells or osteosarcoma cells (MG63) are cultivated under a suitable environment over a pre-fabricated scaffold, and this system of cells and scaffold is then used as a graft [5, 6].

The scaffold is used to provide anchorage and biochemical niche to cells for survival and proliferation. Several properties viz. mechanical strength, osteoinduction, bioresorption, graduated porosity, and biocompatibility are to be considered while selecting material for fabricating scaffold. Osteoinduction (induction of bone formation) is one of the required properties of material to be used in the fabrication of scaffold of bone tissue engineering (BTE) [7]. Scaffolds with osteogenic factors are potent for mimicking the bone tissue regeneration process which couples angiogenesis and osteogenesis which may recruit progenitor cell and its differentiation. Bone morphogenic proteins (BMPs) are the class of growth factors which induces bone formation and are proposed for BTE applications along with de-mineralized bone matrix (DBM) and calcium phosphate [8–10].

Several groups have reported use of metals, ceramics, synthetic polymers and composites, and silk fibroin as potential materials for scaffold fabrication in BTE. Silk fibroin (SF) has been reported as a suitable material for scaffold fabrication for tissue engineering application owing to its remarkable mechanical and biocompatible properties [5]. Till date, no report has been published evaluating the associative benefits of BMPs with SF electrospun nanoscaffolds.

Here, we report fabrication of novel recombinant human bone morphogenic protein-2 (rhBMP2)-conjugated SF electrospun nanofibrous scaffolds. The scaffolds were compared with that of pure SF scaffolds to elucidate the effect of rhBMP2 conjugation on osteoinduction. Cell viability and cell proliferation properties were also measured to establish the potency of the scaffold for new and better bone tissue engineering applications.

## Methods

### Preparation of Aqueous Solutions of SF/BMP2

At first, SF was isolated from the cocoons of silk worm, *Bombyx mori*, as an aqueous solution. The established protocol was followed with slight modifications [11]. Cocoons were boiled in 100 mL of 0.02 M Na_2_CO_3_for 20 min and then rinsed thoroughly with distilled water to remove excess water-soluble sericin and wax. Extracted fibroin was then dissolved in 9 M lithium bromide solution at 60 °C for 4 h and was further dialyzed against water for 4 days. The final concentration was determined by weighing dry matter after drying and was found to be 7% *w*/*v*. This solution was then used after concentrating to different levels by dialysis against 1 L of 25% polyethylene glycol (PEG, 10,000 g mol^−1^) solution at room temperature. Diluted SF aqueous solutions were prepared by diluting with distilled water, and all solutions were stored at 10 °C till further processing. Lyophilized powder of recombinant human bone morphogenic protein-2 (rhBMP2) was dissolved in PBS (pH 3.8). The protein solution was sterilized with 0.22 μm syringe filters and was added as aqueous solution to each fibroin solution with continuous stirring. Glutaraldehyde-mediated crosslinking was employed to associate BMP with fibroin. Briefly, for 10 mL reaction mixture, 5 mL each of 6% silk fibroin and 1% rhBMP2 were cross-linked using 200 μL glutaraldehyde and 40 μL, 12 N HCl as group-activating agent. With this procedure, three solutions were prepared: (i) 3% silk fibroin with no rhBMP2 (SF), (ii) 3% silk fibroin with 0.5% of rhBMP2 (SF+rhBMP2), and (iii) 12% silk fibroin with 0.125% of rhBMP2 as in (ii) (4SF+rhBMP2). These solutions were used in electrospinning procedures for fabrication of scaffold.

### Fabrication of Scaffold by Electrospinning

For scaffold fabrication, each solution was loaded in a 5-mL glass syringe with a stainless steel needle (25G, ID 0.26 mm, Sigma Aldrich) which is connected to a 5.5-kV DC supply. For preparation of fibers, outlet flow rate was maintained at 0.4 mL h^−1^ using a syringe pump, and electrospun fibers were collected on an aluminum foil kept at a gap of 15 cm from capillary tip. The samples were collected for 4 h each.

### Scanning Electron Microscopy

For morphological examination of prepared scaffolds, SEM was performed using Zeiss EVO40SEM. Samples were sputter coated with gold before scanning images were further processed. Determination of fiber diameter is done by averaging the diameters of 10 random fibers in the image frame.

### Mechanical Properties of Scaffold

Compressive experiments were done to assess mechanical properties of developed scaffolds using Instron single column table top electromechanical tester (model 3345, Instron, Canton, MA). Fibers with 0.2 mm diameter, obtained by electrospinning at longer durations, were used to determine the tensile strength and elongation at break from the stress–strain curves at 25 °C and 50% humidity.

### Swelling Study

For measurement of swelling ratio, each formulation was dissolved in PBS (pH 7.4) at 37 °C. Samples were taken out at pre-determined time intervals, and dry weight was measured using an electronic balance. The test was continued till equilibrium weight was reached. The swelling ratio was expressed as below:

$$ \mathrm{Swelling}\  \mathrm{ratio}\left(\%\right)=\frac{W\mathrm{s}-W\mathrm{o}}{W\mathrm{o}} $$

where, *W*_o_ = initial dry weight of the nanofibrous matrices and *W*_s_ = weight of the swollen nanofibrous matrices at each time point.

### Cell Culture

Human mesenchymal stem cells (hMSCs) were used in the present study to evaluate the osteoinductive potential of the fabricated nanoscaffolds. hMSCs were cultured and maintained in DMEM with 10% fetal calf serum and 1% penicillin, at 37 °C in 5% CO_2_ humidified atmosphere till 90% confluence was achieved. Cells were then trypsinized, centrifuged, and re-suspended into medium for quantification.

Scaffolds were sterilized by washing with ethanol and irradiating with UV light for 30 min and washed with PBS (pH 7.4) thereafter. A treatment with DMEM is given to scaffolds before cell seeding. 20 μL of cell suspension was added dropwise to each scaffold and plastic film serving as control. Scaffolds were kept at rest in humidified atmosphere (37 °C, 5% CO_2_) for 30 min. Then, scaffolds were incubated in DMEM for 21 days with regular replenishment of medium every alternate day.

### Cell Adhesion Assay

To assess the adhesion capacity of cells with the scaffold, the numbers of unadhered cells were counted after 1, 3, and 6 h of initial seeding as per the method in the literature with slight modifications [6]. The cell medium was collected and cell count was done with a hemocytometer. The difference between the initial seeding count and number of un-adhered cells were considered as the number of adhered cells. Results were expressed in terms of percent adhesion as per the following equation:

$$ \%\mathrm{Adhesion}=\frac{\mathrm{Initial}\  \mathrm{seeding}-\mathrm{number}\  \mathrm{of}\  \mathrm{non}\  \mathrm{adherent}\kern0.5em \mathrm{cells}}{\mathrm{Initial}\  \mathrm{seeding}}\times 100 $$

### Cytotoxicity Assay

To measure the toxic effect of nanofibrous matrices, MTT assay was performed. After respective time frame, constructs were incubated in MTT solution (1 mg mL^−1^stock solution diluted in PBS (pH 7.4) in a ratio of 1:10) and incubated for 4 h. Viable cells convert MTT into formazan salt during this incubation period. The formazan salt was dissolved by the addition of DMSO and kept aside for 20 min. The absorbance originating from formazan salt was quantitatively measured by recording changes in absorbance at 570 nm using a microplate reader.

### Cell Proliferation Assay

Alamar blue (AB) dye reduction assay was performed to determine proliferation of cells within the scaffold. Scaffolds were incubated in dye diluted with DMEM for 4 h, and reduction in dye was measured spectrophotometrically. Percent AB reduction was calculated as:

$$ \%\mathrm{AB}\ \mathrm{reduction}=\left[\left({\varepsilon}_{\mathrm{ox}}{\lambda}_2\right)\left(\mathrm{A}{\lambda}_1\right)-\left({\varepsilon}_{\mathrm{ox}}{\lambda}_1\right)\left(\mathrm{A}{\lambda}_2\right)/\left({\varepsilon}_{\mathrm{red}}{\lambda}_1\right)\left({\mathrm{A}}^{'}{\lambda}_2\right)-\left({\varepsilon}_{\mathrm{red}}{\lambda}_2\right)\left({\mathrm{A}}^{'}{\lambda}_1\right)\right]\times 100 $$

where, *ελ*_1_ = molar extinction coefficient of alamar blue at 570 nm and *ελ*_2_ = the molar extinction coefficient of alamar blue at 600 nm, in the oxidized (*ε*_ox_) and reduced (*ε*_red_) forms. A*λ*_1_ and A*λ*_2_ denoted the absorbance of test wells.

A’*λ*_1_ = absorbance of negative control well at 570 nm.

A’*λ*_2_ = absorbance of negative control well at 600 nm.

### ALP Assay

Alkaline phosphatase (ALP) production by cultured hMSC within the scaffold was measured as per the manufacturer protocol in the kit [12]. Briefly, sterile PBS (pH 7.4) was used in washing and incubation of scaffolds, followed by homogenization with 1 mL Tris buffer (1 M, pH 8.0), and sonication for 3 min on ice. 25 μL of the lysate was then incubated with 1 mL of p-nitrophenyl phosphate solution (16 mM) at 30 °C for 5 min. Spectrophotometric measurements was performed at 405 nm to monitor the production of p-nitrophenol in the presence of ALP.

### In vivo ALP Activity

Nine male athymic nude rats, weighing 100–120 g each were taken and were dissected bilaterally at the abdominal muscles to create pouches. A nude mouse model was utilized to demonstrate the osteoinductive potential of the scaffold in vivo. One of the each three type of scaffolds (5 mm × 5 mm) were cut and packed into the muscle pouches separately. The pouch was then closed with a non-absorbable suture. After 14 days of operation, implants were retrieved by excising recti abdominal muscles and were kept in PBS. Muscle flaps were excised, and explant tissue is obtained which was homogenized in extraction buffer to release alkaline phosphatase. 50 μL of aliquot of solution was used for the measurement of ALP activity.

### Statistical Analysis

All experiments were done in triplicates, and data presented are formatted as mean ± standard deviation (SD) of samples, unless mentioned. One way analysis of variance (ANOVA) was performed using statistical software Origin 6.0, to evaluate uncertain differences and significant differences. *P* value of 0.05 or less signifies significant differences between the study groups.

## Results

### Morphology of Scaffold

**Fig. 1:**
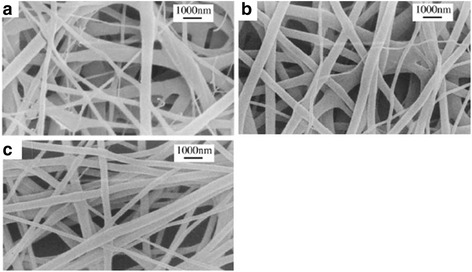
SEM micrographs of prepared scaffolds. **a** SF. **b** SF+rhBMP2. **c** 4SF+rhBMP2

SEM images (Fig. 1) of the fabricated scaffold revealed finely spun nanofibrous structure. The average diameters of fibers in SF and SF+BMP2 scaffolds appear to be similar, ranging from 100 to 900 nm, at all concentrations, as the diameter is found to be a function of time for which electrospinning was done [13], while SF nanofibers were found to be uniform, and BMP2 conjugation led to non-uniformity in fiber diameter. The pore size of the scaffolds appears to be homogenous in the fabricated scaffolds and found to be independent of the concentration of fibroin. The concentration of SF does not affect the pore size significantly [11].

### Mechanical Properties of Scaffold

**Fig. 2:**
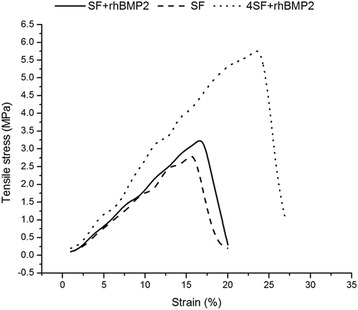
Stress–strain relation of electrospun nanofibers. The stress–strain relationship was compared between (a) SF, (b) SF+rhBMP2, and (c) 4SF+rhBMP2 scaffold

Stress–strain curves of nanofibrous scaffolds are represented in Fig. 2. It was observed that addition of BMP does not alter the mechanical properties of SF scaffold, but with the increase in concentration of fabricating material (SF), tensile property of matrices was improved. It may be due to formation of inter-fiber bonds during crosslinking. Low-concentration SF fibers thus did not exhibit better mechanical strength as compared to higher one.

### Swelling Study

**Fig. 3:**
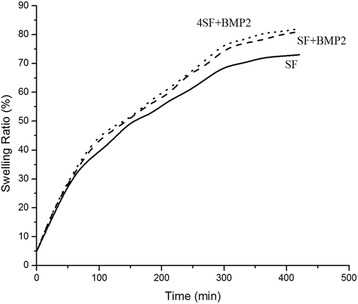
Swelling property of fabricated scaffold. Changes in the swelling property of SF scaffold was observed after the modification with SF+rhBMP2 and 4SF+rhBMP2

Swelling ratios as a function of time for the scaffolds were represented in Fig. 3. The scaffolds swelled well with time uniformly initially and reached to equilibrium in around 380 min. rhBMP2-linked fibers absorbed more water as compared to SF-only scaffolds suggesting the increase in hydrophilic pockets due to BMP2 association. SF fibers equilibrated at ∼ 70% while BMP2 containing fibers were equilibrated at ∼ 81% water.

### Cell Adhesion Assay

**Fig. 4:**
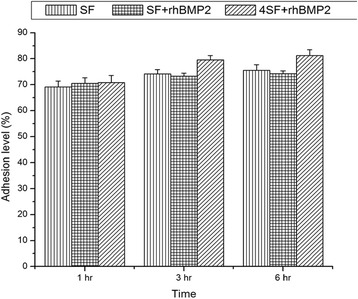
Histogram representing percentage adhesion versus time for three time points. Changes in the adhesion level in (a) SF, (b) SF+rhBMP2, and (c) 4SF+rhBMP2 scaffold

Adherence of cells to the scaffold is required for the growth of cells and induction for differentiation. In this study, we observed that hMSCs adhered well to the scaffolds, and the adherence of hMSC to the 4SF-BMP2, SF-BMP2 and SF scaffold was represented in Fig. 4.

There was a concern of loss in the adherence in the blended scaffold which was ruled out with the observed results. The blending with BMP2 does not decrease the adhering capacity of scaffold. As evident from Fig. 3, it was well understood that with an increase in the pore size (decrease in concentration of SF), adherence of cell to the scaffold increases. ANOVA among the three formulations significantly differentiates variations in the 3rd and 6th hour. However, there was no significant difference observed in the 1st hour.

### Cytotoxicity Assay and Cell Proliferation Assay

**Fig. 5:**
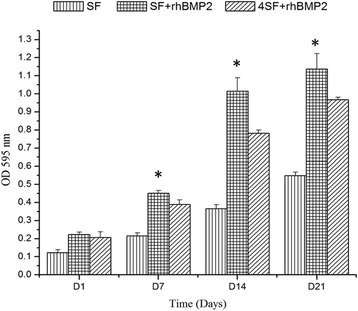
Cell viability assay represented as histogram for four time points. The cell viability assay was performed by MTT assay, and the results are presented as percentage relative to control

Cell viability was significantly increased in the rhBMP2-conjugated scaffolds, and the constructed scaffolds do not create any cytotoxic effects to the guest cells in concern (Fig. 4), and cell proliferated well in all the scaffolds comparatively Fig. 5. There was an increasing trend in viability with number of days, and SF+BMP2 scaffold exhibited least toxicity at each time point. ANOVA revealed significant difference among the values of cell viability of three formulations in concern.

**Fig. 6:**
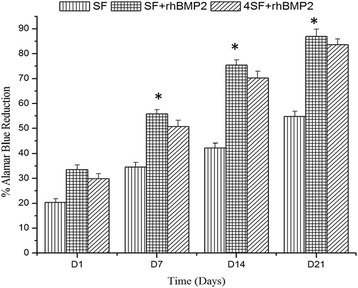
Representation of cell proliferation as percentage of alamar blue dye reduction at four time points for SF, SF+BMP2, and 4SF+BMP2

From Fig. 6, cell proliferation can be examined. Cell proliferated well in all the three scaffold preparations with best in SF+BMP2 each time point. Larger pores in SF + BMP2 scaffold were providing maximum space for growth of cells. The significance of differences among the group was well established from ANOVA.

### ALP Assay

**Fig. 7:**
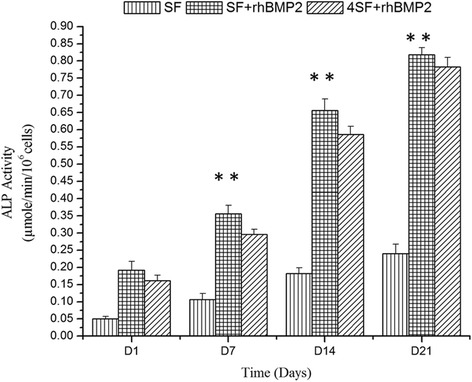
Representation of ALP activity among three different scaffold fabrication strategies at different time points. (a) SF (b) SF+rhBMP2 (c) 4SF+rhBMP2 scaffold

ALP activity is a standard marker of osteoinductive property of the environment around the cell [13]. In our experiment, we observed a higher ALP activity in the SF+BMP2 constructs as compared to SF-only constructs (Fig. 7). SF nanostructured scaffolds were able to exhibit osteoinduction alone as well but as evident from Fig. 7 and ANOVA, SF+BMP2 scaffold proved to be best among the scaffolds in concern. The ALP concentration was increased in due course of time of experiment, and the constructs with higher SF concentration, however, exhibited lower ALP activity in comparison to constructs with lower SF concentration.

### In vivo ALP Activity

**Fig. 8:**
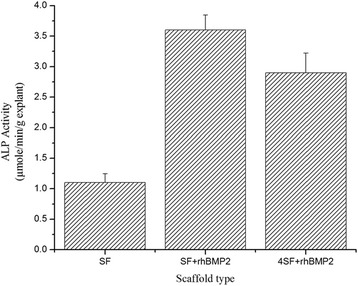
In vivo ALP activity of the explants obtained after treatment. Nude mouse model was utilized to demonstrate the osteoinductive potential of the scaffold in vivo

Fig. 8 depicts ALP activity of explants for (a) SF, (b) SF+rhBMP2, and (c) 4SF+rhBMP2. As expected, explants from treatment containing rhBMP2 induced higher ALP activity while rhBMP2-free scaffold-treated mice produced lower ALP activity.

## Discussions

Scaffold-based tissue engineering has proved its potential in regenerative medicine and has witnessed impressive progress as a tool for BTE. Previous studies have established the crucial role of microarchitecture and physical properties of structures in translating in vitro-engineered cell scaffold constructs into bone tissue [14–16].

Optimum mechanical properties (pore size, tensile strength, etc.) and biocompatibility of the constructs are potential features to be considered for cell colonization and organization [17]. The presented work describes fabrication and characterization of scaffold for bone tissue engineering utilizing combination of beneficial properties of materials proposed for the same. While SF provides a suitably strong and biocompatible platform, embedded rhBMP2 induces the formation of new osteocytes. SF is being extensively studied by several groups in different formulations for osteoblastic cell proliferation [18] and tissue regeneration including ligament, tendon, cartilage, bone, liver, skin, trachea, cornea, nerve, eardrum, and bladder [19, 20].

We have used aqueous solutions of SF and SF+rBMP2 for our study as aqueous solutions are preferred over organic solvents for preparation of SF solutions as degradation of SF is unfavorable in organic solutions [18]. Electrospun SF fibers were previously reported to have homogenous structure of fibers with uniform diameter, and the mesh is highly porous with interlinked and cross-connected pores which is in close accordance with our study as well [21, 22]. There was no observation of formation of bead-like structures in SF fibers in our scaffolds and the previously reported ones [21]. Diameter of the pure SF fiber was previously reported to lessen with increase in blend [11, 21]; however, we did not observe a loss in diameter due to blending. But uniformity of fiber was disturbed in blended fibers possibly due to uneven association of rBMP2 onto the SF fibers.

Ample mechanical strength is a property essential for a tissue scaffold. Blending of pure SF increased the flexibility of nanofiber in our experiment, and previous reports also revealed a similar trend in improvement of mechanical properties upon blending of SF with other materials to yield employable blended biomaterials [21, 23]. So, our fabricated scaffold possesses essential mechanical strength and flexibility which is required in tissue engineering applications.

Scaffolds for tissue engineering should be able to attach cells over it, should facilitate cell proliferation and osteoinduction and should be least cytotoxic for its better acceptability. Earlier reports on SF scaffolds have established its non-cytotoxic and cell-proliferative activities [21, 24], and our studies were in close accordance with the previously reported results.

Owing to its osteoinductive properties, rhBMP2 is being utilized in bone tissue engineering by several groups [25–27]. These studies have revealed that cells cultured over BMP2-containing scaffolds possessed higher ALP activity, a biomarker of osteoinduction. The effect of BMP2 association was better contrasted as the time of incubation progressed. Kim et al. utilized BMP2-associated porous microspheres and observed a similar enhancement in osteoinduction [25].

## Conclusions

We successfully fabricated SF-based fibrous scaffolds containing rhBMP2. These scaffolds were homogenous and were found to have adequate mechanical properties and biocompatibility. Further rhBMP2 association attributed to the osteoinductive potential of the fabricated scaffolds. The scaffolds were further evaluated for in vivo applications and found to be suitable for application involving bone tissue engineering.

## Abbreviations

ALP: Alkaline phosphatase
BTE: Bone tissue engineering
hMSCs: Human mesenchymal stem cells
rhBMP2: Recombinant human bone morphogenic protein-2
SF: Silk fibroin
